# DNAJC8: a prognostic marker and potential therapeutic target for hepatocellular carcinoma

**DOI:** 10.3389/fimmu.2023.1289548

**Published:** 2024-01-11

**Authors:** Zhibo Zhang, Mingxiu Ju, Zhongming Tang, Zhen He, Shengni Hua

**Affiliations:** Department of Radiation Oncology, Zhuhai People’s Hospital, Zhuhai Hospital Affiliated with Jinan University, Zhuhai, China

**Keywords:** DNAJC8, hepatocellular carcinoma, bioinformatics analysis, apoptosis, tumor immune microenvironment

## Abstract

**Background:**

Hepatocellular carcinoma (HCC) is the most common type of liver cancer, accounting for ~90% of the total cases. DnaJ heat shock protein family member C8 (DNAJC8), belonging to the heat shock protein 40 (HSP40) family, is known to regulate cancer biology function. However, the role of DNAJC8 on HCC development remains unknown.

**Methods:**

The Cancer Genome Atlas, GTEx, cBioPortal, and Human Protein Atlas were used to analyze the expression and clinical significance of DNAJC8 in HCC. Two HCC cell lines, MHCC-97H and Huh-7, were utilized to determine the biological function of DNAJC8.

**Results:**

DNAJC8 expression was upregulated in HCC tissues and correlated with poor clinical prognosis. It was closely related to spliceosome, nucleocytoplasmic transport, and cell cycle and might be involved in the formation of tumor immunosuppressive microenvironment. Knockdown of DNAJC8 severely inhibited HCC cell proliferation and induced apoptosis.

**Conclusion:**

Our study demonstrate that *DNAJC8* functions as an oncogene in HCC and hence may be used as a potential therapeutic target and prognostic marker for HCC.

## Introduction

Hepatocellular carcinoma (HCC) is the most common primary liver malignancy, and surgical resection is still the main treatment for it (1). However, due to the concealment of liver cancer, it is often diagnosed at the advanced stage. Systemic treatment is the only effective method for these patients (2). Unfortunately, drugs targeting HCC are mostly multi-target kinase inhibitors, such as sorafenib and lenvatinib (3). Furthermore, individual sensitivity and drug resistance greatly limit their clinical application (4, 5). Therefore, more and more attention are paid to non-kinase target proteins, such as ASCT2, SPR (6, 7). Thus, exploring novel non-kinase treatment targets of HCC is urgently needed.

Heat shock proteins (HSPs) are a type of evolutionarily highly conserved proteins. They are induced by a range of environmental stimuli, especially high temperature, and act as intracellular homeostasis protectors (8). After binding to other proteins, HSP help amino acid chains to fold correctly, eliminating damaged amino acid chains, and avoid cell death (9, 10). The HSP40/DNAJ family is the largest HSP family, containing at least 49 members, which can be divided into three subclasses: DNAJA, DNAJB, and DNAJC (11). Most of the members contain a “J” domain that can bind to HSP70 and activate its ATPase activity to regulate protein folding, unfolding, translation, translocation, and degradation (12). It has increasingly been shown that HSP40/DNAJ family is involved in the regulation of cancer biological functions (13, 14); DNAJA3 can induce apoptosis of breast cancer by regulating p53 (15), and reduce angiogenesis of sarcoma and cervical cancer by destabilizing HIF-1 (16); DNAJB4 arrests lung cancer cell cycle through the STAT1/p21 signaling pathway (17); DNAJB6 inhibits the epithelial mesenchymal transition (EMT) process of breast cancer cells by up-regulating DKK1 and inhibiting Wnt/β-catenin signaling pathway (18); DNAJB1 suppresses p53-dependent apoptosis by destabilizing PDCD5 (19); DNAJC6 can promote the metastasis of HCC via enhancing EMT progression (20). Some studies have shown that DNAJC8 is related to heat tolerance of bee and human spinocerebellar ataxia 3 polyglutamine formation (21). DNAJC8 is also involved in the glycolysis of cervical cancer cells under the regulation of TIG1 (22). However, there is still a lack of research on DNAJC8, especially on its role in cancer.

In this study, through bioinformatics analyses (sample expression, clinical correlation, gene enrichment, and immune infiltration) of data from multiple public databases and *in vitro* cell experiments (siRNA interference), we detected the expression features and function of DNAJC8 and proved that it can serve as an oncogene in HCC. Thus, DNAJC8 may be a potential prognostic and therapeutic target for HCC.

## Materials and methods

### DNAJC8 expression analysis

Paired HCC samples from The Cancer Genome Atlas (TCGA) database (https://portal.gdc.cancer.gov/) were used. The protein expression of DNAJC8 was obtained from UALCAN database (https://ualcan.path.uab.edu/index.html). The immunohistochemical results were obtained from The Human Protein Atlas (www.proteinatlas.org/). Copy number and methylation levels were evaluated using the Liver Hepatocellular Carcinoma (TCGA, PanCancer Atlas) dataset of cBioPotal (www.cbioportal.org/). The genetic variation information was obtained from TCGA Liver Cancer dataset of UCSC XENA and analyzed using the maftools package in R software (3.6.3).

### Clinical prognostic analysis

Survival time and clinical pathological characteristics from TCGA-LIHC datasets were analyzed using the survival (3.2.10) and survminer (0.4.9) packages in R software, respectively. According to the median expression level of DNAJC8, the patients were divided into two groups: high expression group and low expression group. A receiver operating characteristic (ROC) curve was obtained using the pROC package (1.17.0.1). Survival analysis results were obtained from GEPIA (http://gepia.cancer-pku.cn/).

### Enrichment analyses of co-expressed genes

Gene Ontology (GO) (molecular function [MF], cellular component [CC], biological process [BP]) and Kyoto Encyclopedia of Genes and Genomes (KEGG) pathway analyses were performed using the clusterProfiler package (3.14.3) in R software (3.6.3). Gene set variation analysis (GSVA) was done using the GSVA package (1.40.1). Protein–protein interaction network analysis was performed online by STRING database (http://string-db.org).

### Immune infiltration analysis

HCC sample expression data GSE98638 was analyzed online through ImmuCellAI using ssGSEA. TCGA-LIHC expression data (https://portal.gdc.cancer.gov/) was analyzed using estimate (1.0.13) package in R software (3.6.3) via ssGSEA method. Immune checkpoint analysis was performed online using the “correlation analysis” function of GEPIA.

### Immunohistochemistry

11 pairs of HCC tissues fixed with 4% paraformaldehyde were dehydrated and paraffin-embedded and then sectioned. The sections were put into an oven to dry at 63 degrees for 1 hour. Dewaxing was performed with LEICAST5020 (Dako). After antigen repair was completed, the sections were incubated with DNAJC8 antibody at 4 degrees overnight. Blocking, secondary antibody binding, and DAB chromogenic staining was performed with Autostainer Link 48 (Dako). After 1 minute of hematoxylin staining, the sections were immersed in 0.25% hydrochloric acid alcohol for 10 seconds and washed with water for 5 minutes. After sealing with neutral resin, the sections were photographed. The score standard for the intensity of staining was as follows: 0, negative; 1, weak; 2, medium; 3, strong. The extent of staining was scored as: 0, 0%; 1, 1–25%; 2, 26–50%; 3, 51–75%; 4, 76–100%.

### Cell culture

All cell lines in the experiments were obtained from Procell Life Science & Technology Co., Ltd. MHCC-97H and Huh-7 cells were cultured using Dulbecco’s modified Eagle medium (Gibco, Waltham, MA, USA) with 10% fetal bovine serum (Gibco) in a 37°C incubator with 5% CO_2_ (Thermo Fisher Scientific, Waltham, MA, USA).

### siRNA transient transfection

Cells in rapid growth phase were collected and plated in 6-well plates at a density of 200,000 cells per well. After 12 h of incubation, the mixture containing 5 µL siRNA for DNAJC8 (RiboBio, Guangzhou, China) and 5 µL RNAiMAX (Invitrogen, Carlsbad, CA, USA) was added to every well. The cells were cultured in a 37°C incubator with 5% CO_2_ for 48–72 h for further analysis. the siDNAJC8-1 sequence was ‘GATTGAAGCTCAAGAAAAA’; the siDNAJC8-2 sequence was ‘GCAGTTATCCATCTTGGTG’.

### qRT-PCR

Total RNA was isolated from the cell lines using the RNA-Quick Purification Kit (Yishan, Shanghai, China). Approximately 1 μg RNA was reverse-transcribed into cDNA, using the HiScript III RT SuperMix (Vazyme, Nanjing, China), and qRT-PCR was performed using the AceQ Universal SYBR qPCR Master Mix (Vazyme). GAPDH was used as internal control. The primer sequence of *DNAJC8* was that the forward sequence was ‘CCAAACGGGAAAGAGAGTGGCA’; the reverse sequence was ‘ACTTTCGGTGGTCTCAGGAAGG’.

### Western blotting

Western blotting was performed according to our previous reports. The DNAJC8 antibody (ab138506) was obtained from Abcam (1:1000; Abcam, Cambridge, UK). The Bax (50599-2-lg), P53 (10442-1-AP), DNAJB1 (13174-1-AP), Hsp70 (10995-1-AP) and Hsp90 (13171-1-AP) antibody was obtained from Proteintec (Chicago, USA).

### Cell Counting Kit-8 assay

Cells were plated in 96-well plates at a density of 3000 cells per well, and CCK8 solution (Dojindo, Kumamoto, Japan; 10 μL/well) was added at 12, 24, 48, and 96 h. The mixture was incubated at 37°C for 2 h, and the absorbance at 450 nm wavelength was recorded (Thermo Fisher Scientific, Waltham, MA, USA).

### Colony formation

Cells were plated in a 6-well plate at a density of 2000 cells per well. When the clone was formed, the wells were fixed with 4% paraformaldehyde for 2 h, washed with phosphate-buffered saline, and stained with crystal violet for 24 h.

### 5-Ethynyl-20-deoxyuridine assay

The cells were plated in a 96-well plate at a density of 3000 cells per well. Proliferating cells were examined using the Cell-Light EdU Apollo488 *In Vitro* Kit (RiboBio), according to the manufacturer’s protocol.

### Apoptosis analysis

The cells were plated in a 6-well plate at a density of 200,000 cells per well. Annexin V-FITC/PI Apoptosis Detection Kit (Vazyme) were used to analyze the programmed cell death. All operations were carried out according to the manufacturer’s protocol.

### Statistical analysis

Data are presented as the mean ± standard error of the mean of at least three independent experiments. All statistical analyses were performed using SPSS software (Abbott Laboratories, Chicago, IL, USA). The Student*’*s *t*-test was used to determine the significance between groups. Comparisons among multiple groups were analyzed using one-way analysis of variance (ANOVA) and Dunnett’s multiple comparisons. For CCK-8 results, a multi-way ANOVA was adopted. Two-sided *p*-values were calculated, and different numbers of asterisks indicate different levels of statistical significance (*p< 0.05, **p< 0.01, and ***p< 0.001).

## Results

### DNAJC8 expression is upregulated in HCC

**Figure 1:**
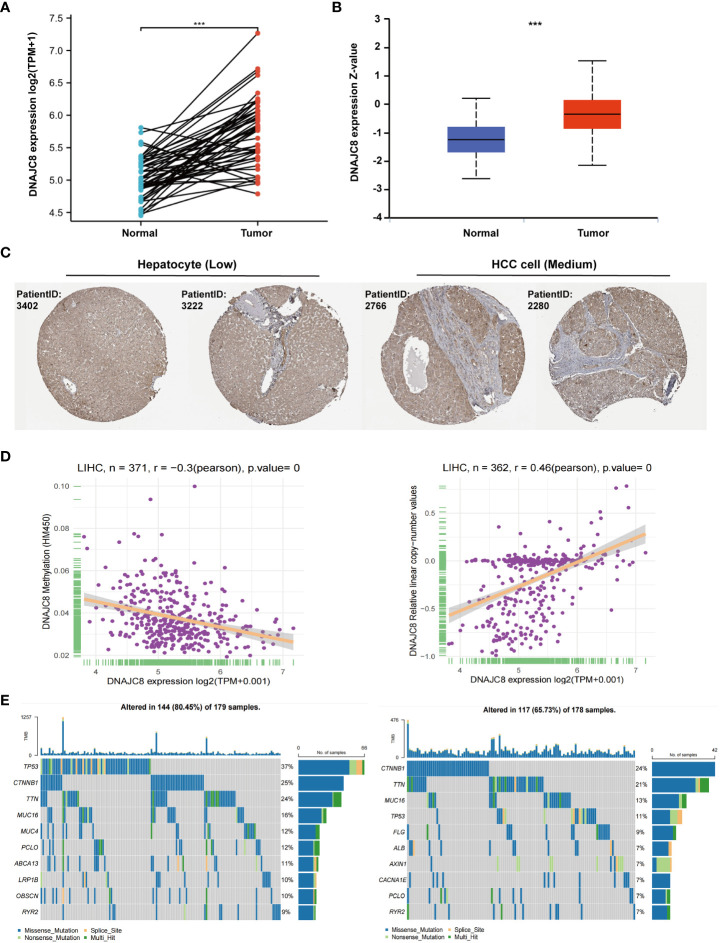
DNAJC8 expression is upregulated in HCC. **(A)** mRNA expression of DNAJC8 in paired samples from TCGA. **(B)** Protein expression of DNAJC8 between normal and tumor tissue from UALCAN. **(C)** Expression difference of DNAJC8 in Human Protein Atlas database. **(D)** The respective relationship between DNAJC8 expression and methylation (left), copy number (right). **(E)** Relationship between gene mutation and DNAJC8 expression (left: high; right: low). ***: *p*<0.001.

TCGA cohort data showed that the DNAJC8 mRNA level in HCC tissues was considerably increased than that in adjacent or normal tissues (**Figure 1A**). DNAJC8 protein expression was upregulated in HCC tissues compared to that in normal liver tissues, according to UALCAN (**Figure 1B**) and Human Protein Atlas database (**Figure 1C**). DNAJC8 expression was negatively correlated with promoter methylation level (r=-0.3, *p*<0.0001) but positively related to gene copy number (r=0.46, *p*<0.0001) (**Figure 1D**). Notably, 37% of patients with high DNAJC8 expression had TP53 mutations, while CTNNB1 mutations were the most common in patients with low DNAJC8 expression (**Figure 1E**; **Supplementary Figure 1A**). These results suggested that DNAJC8 expression was higher in HCC and it may serve as an oncogene.

### DNAJC8 expression is closely associated with HCC prognosis

**Figure 2:**
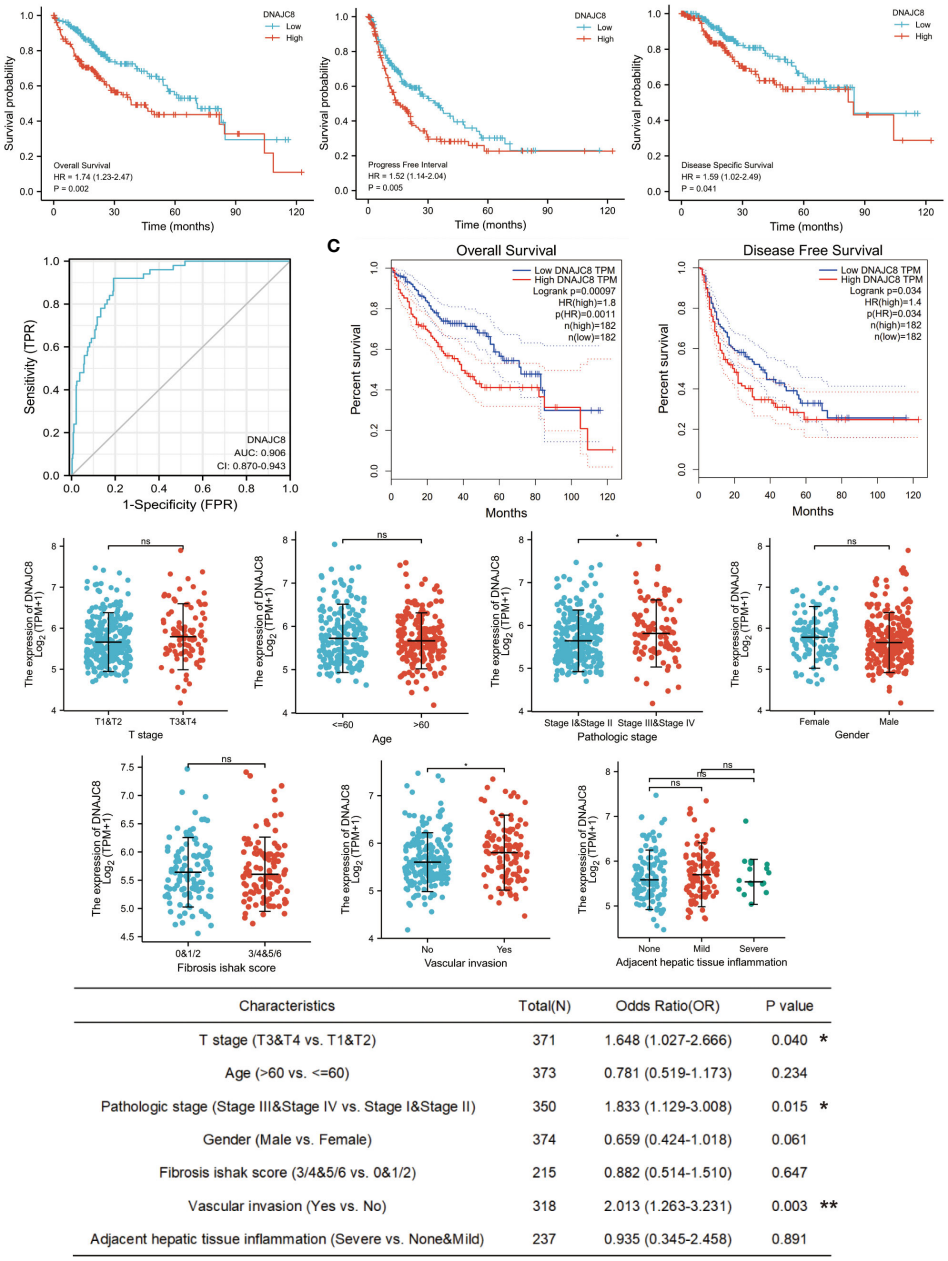
DNAJC8 expression is closely associated with HCC prognosis. **(A)** Survival analysis of DNAJC8 expression (left: overall survival; middle: progression-free survival; right: disease-specific survival) in TCGA. **(B)** The ROC curve of DNAJC8. **(C)** Survival analysis of DNAJC8 expression in GEPIA. **(D)** Relationship between DNAJC8 expression and clinical characteristics. **(E)** Logistics regression analysis between DNAJC8 and clinical characteristics. *: *p*<0.05; **: *p*<0.01.

Survival analysis showed that HCC patients with higher DNAJC8 levels showed significantly poor prognosis in overall survival (HR=1.74, *p*<0.005), progression-free survival (HR=1.52, *p*<0.05), and disease-specific survival (HR=1.59, *p*<0.05) in TCGA cohort (**Figure 2A**). The ROC curve also indicated that DNAJC8 expression could distinguish tumors from non-tumors (AUC=0.906) (**Figure 2B**). Survival analysis results from GEPIA database verified the above results (**Figure 2C**). Furthermore, there was an upward trend of DNAJC8 expression in patients with advanced stage tumor (*p*<0.05) and vascular invasion (*p*<0.05) (**Figure 2D**). Logistics regression analysis confirmed that the expression level of DNAJC8 was correlated with T stage (*p*<0.05), pathological stage (*p*<0.05), and vascular invasion (*p*<0.005) (**Figure 2E**). Therefore, DNAJC8 can be used as an independent prognostic indicator for HCC patients.

### Enrichment analysis of DNAJC8-related genes

**Figure 3:**
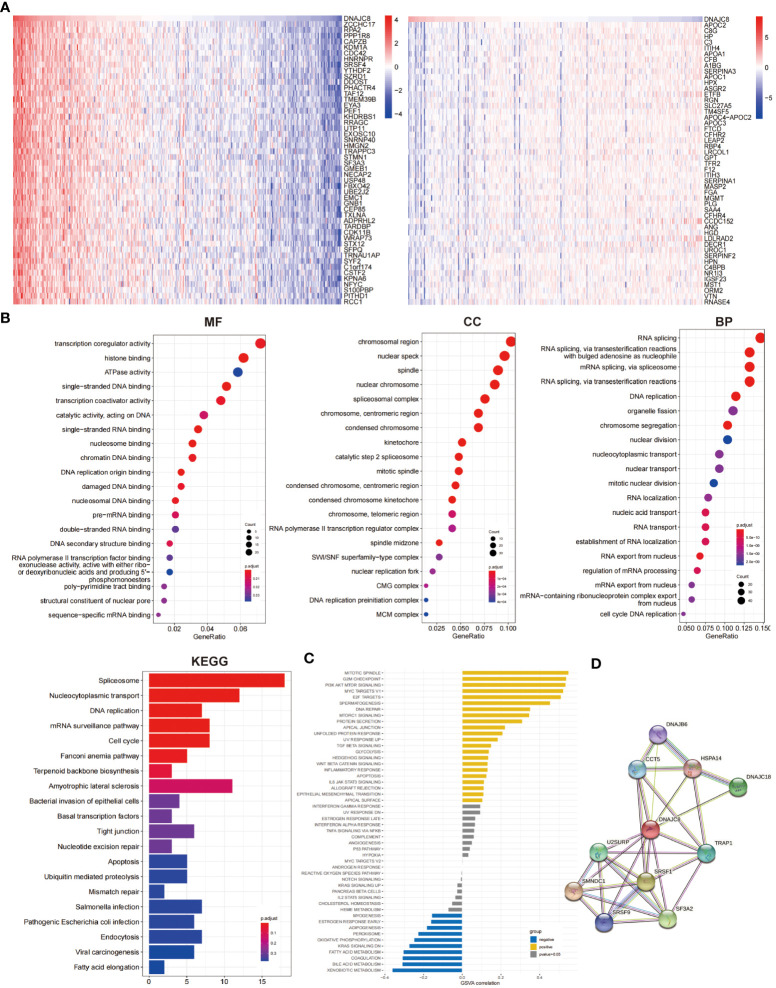
Enrichment analysis of DNAJC8-related genes. **(A)** The heat map of DNAJC8-related genes (left: positive correlation; right: negative correlation). **(B)** GO and KEGG analysis about DNAJC8-related genes. **(C)** GSVA analysis of DNAJC8-related genes. **(D)** PPI analysis of DNAJC8.

In order to identify the genetic alterations and enriched biological functions mediated by DNAJC8, we first picked out the genes significantly correlated with DNAJC8 and found differentially expressed genes, including *zcchc17* (r=0.85, *p*<0.01), *rpa2* (r=0.82, *p*<0.01), *ppp1r8* (r=0.83, *p*<0.01), *capzb* (r=0.80, *p*<0.01), *kdm1a* (r=0.77, *p*<0.01), *cdc42* (r=0.82, *p*<0.01), *hnrnpr* (r=0.80, *p*<0.01), *srsf4* (r=0.79, *p*<0.01), *ythdf2* (r=0.76, *p*<0.01), *szrd1* (r=0.75, *p*<0.01), *apoc2* (r=-0.41, *p*<0.01), *c8g* (r=-0.34, *p*<0.01), *hp* (r=-0.38, *p*<0.01), *c3* (r=-0.37, *p*<0.01), *itih4* (r=-0.36, *p*<0.01), *APOA1* (r=-0.33, *p*<0.01), *CFB* (r=-0.28, *p*<0.01), *A1BG* (r=-0.32, *p*<0.01), *SERPINA3* (r=-0.21, *p*<0.01), *APOC1* (r=-0.42, *p*<0.01) (**Figure 3A**). We then conducted enrichment analyses in terms of MFs, CCs, and BPs and KEGG pathways analyses (**Figure 3B**). GSVA correlation analysis was performed (**Figure 3C**). They all showed that the signaling pathways associated with DNAJC8 were spliceosome (KEGG, *p*<0.01), nucleocytoplasmic transport (KEGG, *p*<0.01), DNA replication (KEGG *p*<0.01), mRNA surveillance pathway (KEGG, *p*<0.01), cell cycle (KEGG, *p*<0.01), mitotic spindle (GSVA, r=0.54, *p*<0.01), G2M_checkpoint (GSVA, r=0.53, *p*<0.01), PI3K_Akt_mTOR_signaling (GSVA, r=0.53, *p*<0.01), MYC_targets_V1 (GSVA, r=0.52, *p*<0.01), E2F_targets (GSVA, r=0.50, *p*<0.01), spermatogenesis (GSVA, r=0.45, *p*<0.01), fatty_acid_metabolism (GSVA, r=-0.30, *p*<0.01), coagulation (GSVA, r=-0.30, *p*<0.01), bile_acid_metabolism (GSVA, r=-0.31, *p*<0.01), and xenobiotic_metabolism (GSVA, r=-0.36, *p*<0.01). Finally, through the string database analysis, we found that DNAJB6 (score=0.746), CCT5 (score=0.782), HSPA14 (score=0.732), DNAJC18 (score=0.860), U2SURP (score=0.839), TRAP1 (score=0.811), SMNDC1 (score=0.940), SRSF1 (score=0.929), SRSF9 (score=0.751), and SF3A2 (score=0.885) may have protein-protein interactions with DNAJC8 (**Figure 3D**; **Supplementary Figure 1B**).

### Abnormal expression of DNAJC8 affects tumor immune microenvironment

**Figure 4:**
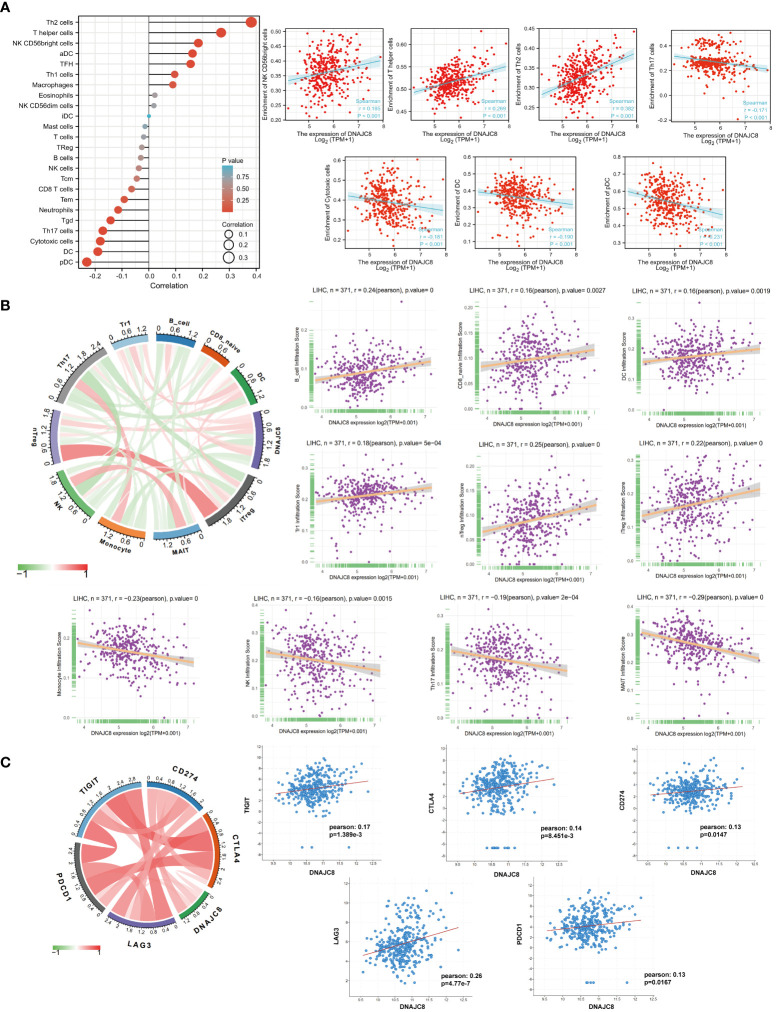
Abnormal expression of DNAJC8 affects tumor immune microenvironment. **(A)** Immune infiltration analysis using GSE datasets. **(B)** Immune infiltration analysis using TCGA datasets. **(C)** Relationship between DNAJC8 and immune checkpoints.

The composition or function of stromal cells, especially immune cells in tumor microenvironment can affect cancer progression. GSE datasets analysis showed that the expression of DNAJC8 was positively correlated with the enrichment of NK CD56 bright (r=0.185, *p*<0.001), T helper (r=0.269, *p*<0.001), and Th2 cells (r=0.382, *p*<0.001), while negatively correlated with the enrichment of Th17 cells (r=-0.171, *p*<0.001), killer toxic cells (r=-0.181, *p*<0.001), DC cells (r=-0.190, *p*<0.001), and pDC cells (r=-0.231, *p*<0.001) (**Figure 4A**). TCGA data analysis revealed that DNAJC8 expression was positively correlated with the infiltration of B cells (r=0.24, *p*<0.0001), CD8_naive (r=0.16, *p*<0.005), DC (r=0.16, *p*<0.005), Tr1 (r=0.18, *p*<0.001), nTreg (r=0.25, *p*<0.0001), and iTreg cells (r=0.22, *p*<0.0001), while negatively correlated with the infiltration of monocytes (r=-0.23, *p*<0.0001), NK (r=-0.16, *p*<0.005), Th17 (r=-0.19, *p*<0.001), and MAIT cells (r=-0.29, *p*<0.0001) (**Figure 4B**). In addition, DNAJC8 levels was positively correlated with the expression of immune molecular checkpoint TIGIT (r=0.17, *p*<0.01), CTLA4 (r=0.14, *p*<0.01), CD274 (r=0.13, *p*<0.05), LAG3 (r=0.26, *p*<0.0001), and PDCD1 (r=0.13, *p*<0.05) (**Figure 4C**). These data indicated that DNAJC8 may be involved in the formation of tumor immunosuppressive microenvironment.

### DNAJC8 knockdown inhibits HCC cell proliferation and induces apoptosis

**Figure 5:**
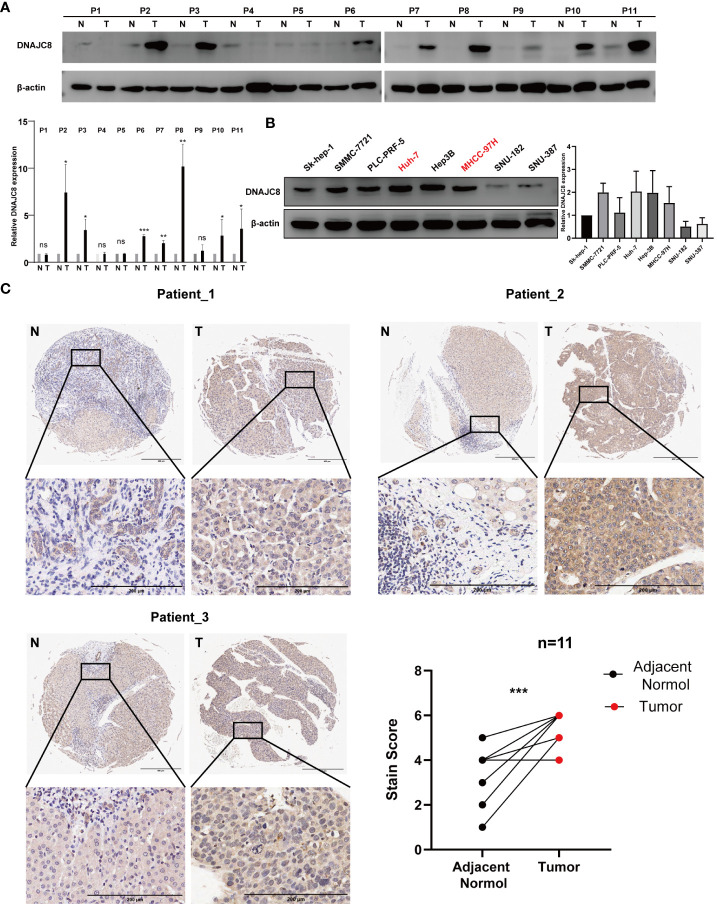
DNAJC8 expression in patients and cell lines. **(A)** Expression of DNAJC8 in paired samples from HCC patients. **(B)** Expression of DNAJC8 in HCC cell lines. **(C)** Immunohistochemical staining of HCC patient tissues (n=11). *: *p*<0.05; **: *p*<0.01; ***: *p*<0.001.

**Figure 6:**
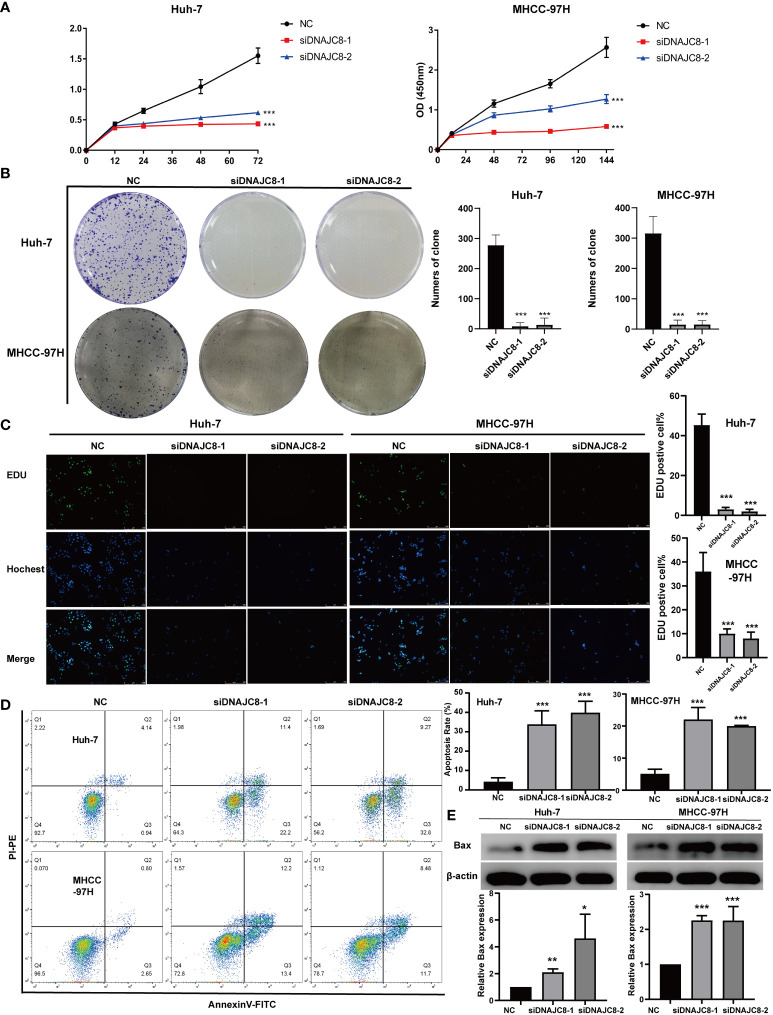
DNAJC8 knockdown inhibits HCC cell proliferation and induces apoptosis. **(A)** Growth curve of Huh-7 and MHCC-97H. **(B)** Colony formation assay of Huh-7 and MHCC-97H. **(C)** EDU assay of Huh-7 and MHCC-97H. **(D)** Apoptosis analysis of Huh-7 and MHCC-97H. **(E)** The up-regulation of Bax caused by the knock down of DNAJC8. *: *p*<0.05; **: *p*<0.01; ***: *p*<0.001.

Consistent with the above results of TCGA, DNAJC8 expression in tumor tissues was much higher than that in adjacent tissues in seven pairs of HCC patients’ samples (**Figure 5A**). Meanwhile, immunohistochemical result further confirmed the upregulated DNAJC8 expression in the tumor tissues (**Figure 5C**). In order to explore the biological roles of DNAJC8, among the nine HCC cell lines, MHCC-97H and Huh-7 with relatively high DNAJC8 expression were selected for subsequent functional experiments (**Figure 5B**). DNAJC8 was successfully knockdown as shown in **Supplementary Figures 1C, D**. The proliferation ability of HCC cells with inhibited DNAJC8 expression was considerably impaired according to CCK-8 assays (**Figure 6A**), clone formation assays (**Figure 6B**), and EdU assays (**Figure 6C**). Furthermore, interference with DNAJC8 expression can induce apoptosis in HCC cells (**Figure 6D**). The expression of pro-apoptotic protein Bax was upregulated after DNAJC8 knockdown (**Figure 6E**). These indicated that DNAJC8 could promote the proliferation and inhibit apoptosis of HCC cells.

## Discussion

DNAJC8, a member of HSP40 family, reports about its functional effects especially in tumor are scarce. Our study found that DNAJC8 expression was upregulated in HCC and has prognosis implications, indicating it may mediate the regulation of biological functions in HCC. HSP40 binds to HSP70 and activates its ATPase activity, which is mainly performed by His-Pro-Asp (HPD) motif in the conserved J region (23). Except for the conserved J region, there is no sequence similarity between DNAJCs and DNAJAs/DNAJBs (24). Hence, it is difficult to predict the function of DNAJCs according to DNAJAs and DNAJBs. Based on the context, it is necessary to analyze the function of DNAJC8 using bioinformatics analyses.

Analysis of TCGA data showed that HCC patients with higher expression of DNAJC8 have higher TP53 mutation frequencies. The relationship between HSP40 family and TP53 has been extensively verified. DNAJA1/HDJ2 directly binds to mutant TP53 (R175H, C176F) to prevent its ubiquitin-proteasome degradation (25). DNAJA3/Tid1 can bind to wild-type or mutant TP53, promoting its mitochondrial translocation, and thus inducing apoptosis (26). DNAJC7 can bind to the DNA binding region of TP53, stabilizing TP53 and further activating it to promote apoptosis (27). However, DNAJC2/ZRF1 promotes tumor development by inhibiting the function of wild-type TP53 (28). Thus, TP53 pathway most likely participate in the mechanistic regulation of DNAJC8. Moreover, DNAJC8 co-expressed gene sets analysis showed these significantly correlated genes were mainly involved in the regulation of chromatin and spindle regions, and their functions were mostly related to DNA binding. GO and KEGG analyses showed that DNAJC8 was associated with DNA replication, spindle function, and chromosome segregation. GSVA analysis showed a strong positive correlation between DNAJC8, mitotic spindle and G2/M checkpoint. These analyses suggest that the abnormal expression of DNAJC8 is associated with cell cycle. Combined with the fact that TP53 is an important regulatory protein in G2/M phase (29, 30), we hypothesized that DNAJC8 regulates HCC cell proliferation mediated by the TP53 pathway. However, we detected the expression of P53 protein after DNAJC8 knockdown, but no significant difference was found (**Supplementary Figure 2B**). According to the Cellosaurus database (https://www.cellosaurus.org/), both Huh-7 (c.659A>G) and MHCC-97H (c.151G>T) have P53 mutation, so it may not be appropriate to explore the regulatory relationship between DNAJC8 and P53 using these two cell lines. We will use HCC cells with different states of P53 in the future to explore the detailed regulatory mechanism.

The relationship between HSP40 family and tumor immune microenvironment remains unclear. Thus, exploring the interaction between DNAJC8 and immune cell infiltration will help us further understand the underlying mechanism of DNAJC8. Our analysis verified that the infiltration of killer cells (mononuclear-macrophages, NK, CD8+T, and MAIT cells) decreased, while the infiltration of helper cells (Th2, Tr1, nTreg, iTreg, NK CD56 bright), playing an immunomodulatory role and inhibiting the function of killer cells, increased in HCC tissues with higher DNAJC8 expression. These indicate that DNAJC8 is associated with tumor immunosuppressive microenvironment. However, the infiltration trend of DC cells was not consistent between the two databases analyses. Therefore, further verification in the follow-up study is needed. In addition, immune checkpoint molecular expression analysis also confirmed that five immune checkpoint molecular were significantly positively correlated with DNAJC8 expression, suggesting that DNAJC8 may promote tumor immune escape through checkpoint pathway. Therefore, the high expression of DNAJC8 is significantly related to immunosuppression microenvironment of cancer and detailed experimental investigation is needed in the future.

In order to investigate the function of DNAJC8, we used siRNA technology to knockdown DNAJC8 in MHCC-97H (TP53:c.151G>T) and Huh-7 (TP53:c.659A>G) cell lines. The results demonstrate that cell proliferation was inhibited and apoptosis was induced. In fact, clinical correlation analysis indicated that DNAJC8 expression was closely related to vascular invasion, and the HSP40 family had been proved to play an important role in tumor metastasis. Zhang et al. found that DNAJB6 promote rectal cancer cell invasion through IQGAP1/erk signaling pathway (31). It has been reported that DNAJA1 can stabilized the expression of EF1A1 by binding *miR-205-5p* to enhance the metastasis progress (32). Therefore, DNAJC8 is also likely to be involved in modulation of cancer metastasis. As DNAJC8 knockdown significantly injured cells *in vitro*, migration-related experiments were not explored.

## Conclusions

DNAJC8 expression is upregulated in HCC and can serve as a prognostic indicator for HCC. DNAJC8 promotes the proliferation and inhibits apoptosis of HCC cells and interferes with the tumor immune response. Undoubtedly, DNAJC8 is worthy of further exploration as a therapeutic target for HCC.

## Data availability statement

The datasets presented in this study can be found in online repositories. The names of the repository/repositories and accession number(s) can be found in the article/**Supplementary Material**.

## Ethics statement

The studies involving humans were approved by the institutional review board of Zhuhai Hospital Affiliated with Jinan University. The studies were conducted in accordance with the local legislation and institutional requirements. The participants provided their written informed consent to participate in this study.

## Author contributions

ZZ: Writing – original draft, Data curation, Methodology, Software. MJ: Writing – original draft, Validation. ZT: Writing – original draft, Software. ZH: Writing – original draft, Investigation. SH: Writing – original draft, Writing – review & editing, Funding acquisition, Project administration, Supervision.
