# Supplementary material for: DNAJC8: a prognostic marker and potential therapeutic target for hepatocellular carcinoma
Source: Front Immunol. 2024 Jan 11;14:1289548. doi: 10.3389/fimmu.2023.1289548 (PMC10808467; doi:10.3389/fimmu.2023.1289548)
Supplement: Supplementary file 1 [file DataSheet_1.docx]

Supplementary Material

## Supplementary Figures


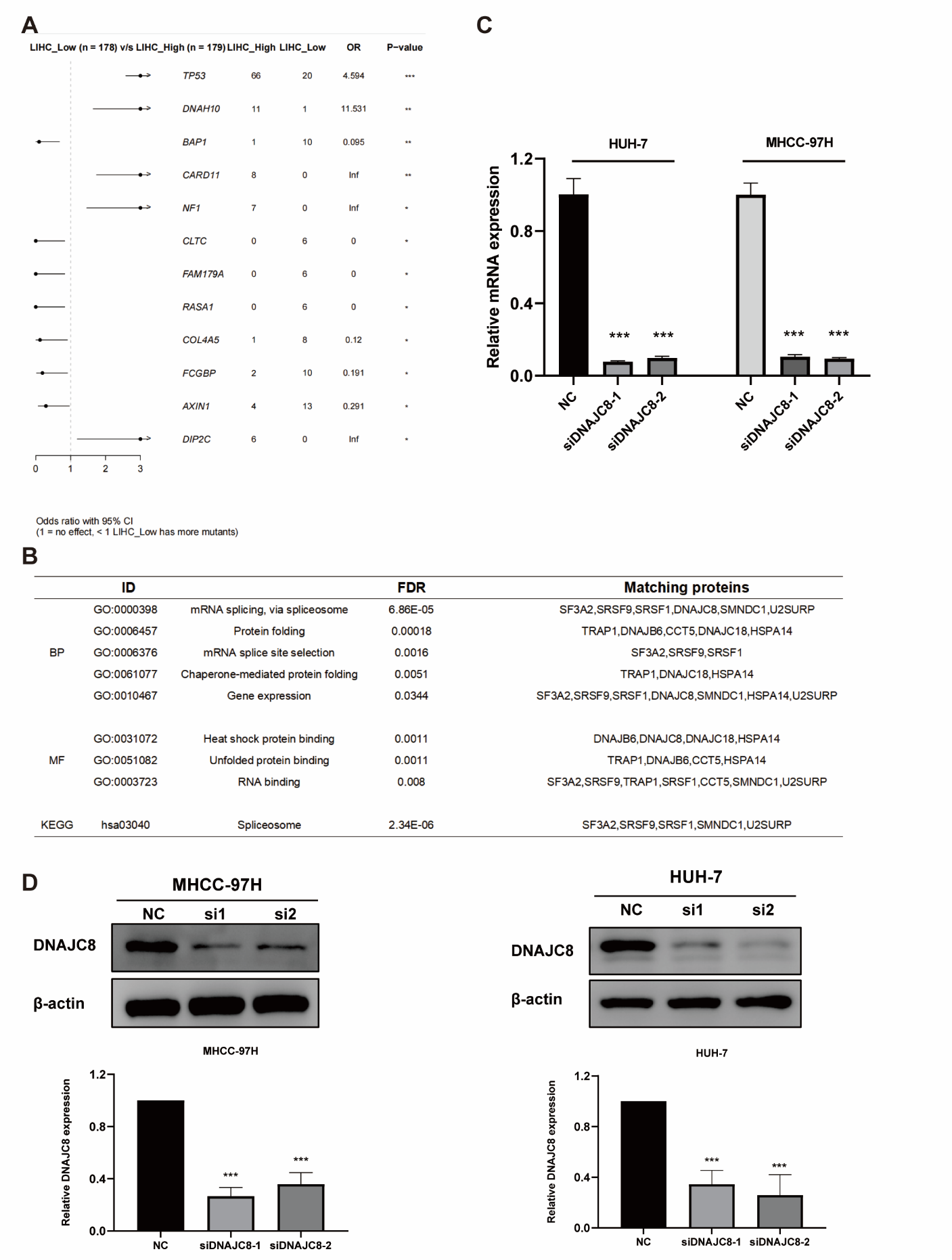


**Supplementary Figure 1.** **(A)** The mutant gene associated with DNAJC8 expression. **(B)** The GO analysis of DNAJC8-related proteins. **(C)** Knockdown efficiency of siRNA in mRNA expression. **(D)** Knockdown efficiency of siRNA in protein expression. * : *p*<0.05; ** : *p*<0.01; *** : *p*<0.001.


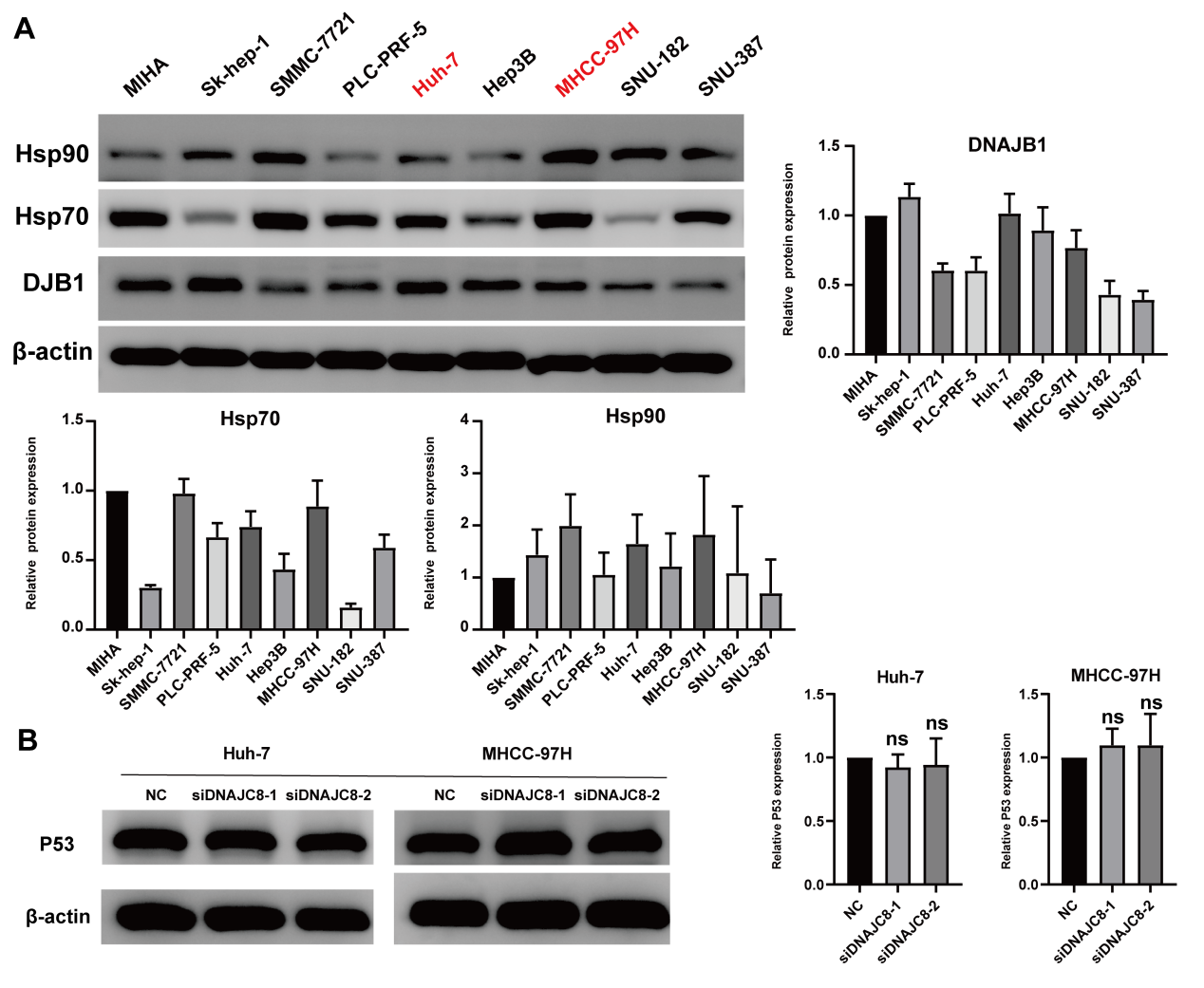


**Supplementary Figure 2. (A)** Expression of DNAJB1, Hsp70, Hsp90 in cell lines. **(B)** P53 expression after DNAJC8 knocdown.

## Raw Data

**Please visit** https://www.jianguoyun.com/p/DYXndp0Q6Nz-CxiJkJ4FIAA **to view the raw data.**
